# Changes in the epidemiological characteristics of prehospital emergency services before and during the COVID-19 pandemic, Chengdu, 2016–2021

**DOI:** 10.1038/s41598-023-34852-y

**Published:** 2023-05-13

**Authors:** Bihua Zhang, Wenchao Kang, Shiqiang Xiong, Xia Huang, Pei Chen, Jinmin Huang, Yufei Hou, Lin Ma, Tao Xiang

**Affiliations:** 1 Institute of Biomedical Engineering, College of Medicine, Southwest Jiaotong University, Chengdu, China; 2 Department of Emergency, The Affiliated Hospital of Southwest Jiaotong University, The Third People’s Hospital of Chengdu, No. 82 Qinglong Street, Chengdu, Sichuan China; 3 Chengdu Medical Emergency Center, Chengdu, China; 4 Department of Cardiology, The Affiliated Hospital of Southwest Jiaotong University, The Third People’s Hospital of Chengdu, Chengdu, China

**Keywords:** Medical research, Epidemiology, Public health

## Abstract

The coronavirus disease 2019 (COVID-19) brought a healthy crisis all around the world. It’s not only affected healthcare utilization, but also some disease’s incidence. We collected pre-hospital emergency data in Chengdu from January 2016 to December 2021, and studied the demand for emergency medical services (EMSs), emergency response times (ERTs), and the disease spectrum in the city proper of Chengdu as a whole. A total of 1,122,294 prehospital emergency medical service (EMS) instances met the inclusion criteria. Under the influence of COVID-19, notably in 2020, the epidemiological characteristics of prehospital emergency services in Chengdu were considerably altered. However, as the pandemic was brought under control, they returned to normal or even earlier in 2021.The COVID-19 pandemic had an effect on people's medical care-seeking behavior and the performance of prehospital emergency services. Although the indicators associated with prehospital emergency services eventually recovered as the epidemic was brought under control, they remained marginally different from before the outbreak.

## Introduction

The World Health Organization declared COVID-19 a pandemic on March 11, 2020. The illness was diagnosed for the first time in December 2019 in Wuhan, China. According to the statistics of the World Health Organization, there have been 635,962,174 confirmed cases of COVID-19 and 6,613,086 deaths worldwide^1^. In reaction to this unprecedented virus, many countries have implemented extremely stringent epidemic prevention measures; thus, COVID-19 greatly affected the utilization and expenditure of health care services as well as the incidence of some diseases^2–9^. Furthermore, these measures also influenced the EMS calls and dispatches, international transmission of COVID-19, the performance of prehospital EMS personnel, people’s life expectancy, people's willingness to seek medical treatment, etc.^10–14^. According to research conducted in Scotland, accident and emergency visits and emergency hospital admissions have decreased as a result of COVID-19. Despite recovery, visit rates remained below prepandemic levels^15^. Another study conducted at a trauma center in Louisville found that the epidemic drastically reduced the number of emergency department visits, which did not return to prepandemic levels^11^. A study conducted in Tehran, Iran revealed that the volume of EMS calls and dispatches increased due to the impact of COVID-19^13^. However, there is currently a lack of research on the long-term effects of this epidemic on the pre-hospital emergency system, particularly in major cities in the southwestern region of China.

Prehospital emergency care is a vital component of the prehospital EMS system and is also an important measure of the overall medical service level^16^. In addition to clinical care, personnel, the environment, resources, and logistics all have an impact on prehospital emergencies^17^. Previous research has shown that the COVID-19 pandemic had an impact on the pre-hospital emergency system, with some studies reporting a decrease in emergency department visits in certain countries, while others have found an increase in EMS calls and dispatches^11,13,15,18^.This indicates that differences in epidemic prevention policies, public awareness of the epidemic, and pre-hospital EMS model could all potentially affect the utilization of pre-hospital emergency services.

In the context of recurrent outbreaks of public health emergencies, it is crucial for relevant departments to develop an emergency plan to coordinate medical resources, guide people to seek medical treatment correctly, and prevent the deterioration of patients' illnesses, which might cause serious consequences. Drawing lessons from past events can provide a foundation for establishing a more advanced and superior urban emergency medical service system. Therefore, the purpose of this study was to determine how the COVID-19 pandemic has altered the epidemiological characteristics of prehospital emergency services in a megacity with a population of more than 13 million people.

## Results

**Figure 1:**
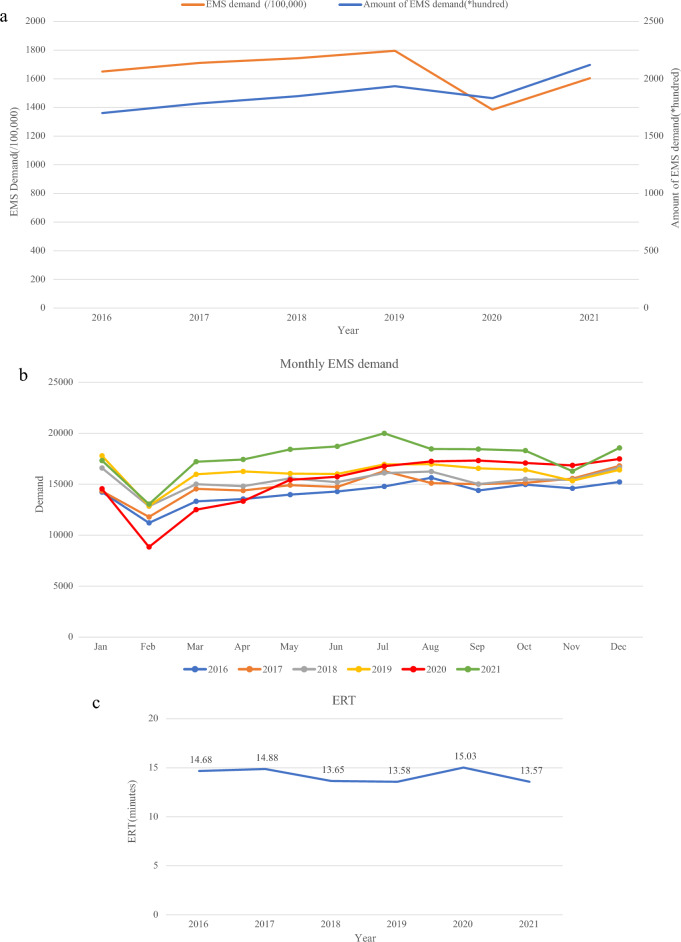
(**a**) Cases of pre-hospital amount of EMS demand and EMS demand per 100,000 population and changing trends, (**b**) The monthly EMS demand in 2016–2021, (**c**) Changes in ERT.

A final total of 1,122,294 records between 2016 and 2021 were included in our study. Figure 1a shows the number of EMS demands. In comparison, demand grew steadily before the pandemic and declined substantially in 2020 after the pandemic broke out but increased again in 2021 after the pandemic was brought under control. Furthermore, by analyzing the demand for first aid in each month of the year, we found that after the government activated level I response to public health emergencies, the demand for EMS declined sharply and rebounded slowly although the government had implemented regular epidemic prevention and control measures (Fig. 1b). From 2016 to 2021, the number of valid dispatches and dispatches had the same changing trend as the demand. Furthermore, the effective dispatch rate grew smoothly over the course of the pandemic (Table 1). The ERT was 13.58 min in 2019, increased to 15.03 min in 2020, and decreased again to 13.57 min in 2021 ($p<0.001$) (Fig. 1c).

**Table 1:** Analysis of the command dispatch situation of pre-hospital emergency in 2016–2021. Values are the number of dispatch ambulances and valid dispatch ambulances; The valid dispatch ambulances refer to the ambulances that received patients after emergency center assigns and completed their treatment and transfer tasks; Effective dispatch = The valid dispatch ambulances/ the dispatch ambulances.

Year	The dispatch ambulances	The valid dispatch ambulances	Effective dispatch (%)
2016	179,448	159,576	88.93
2017	189,535	168,628	88.97
2018	199,703	174,892	87.58
2019	206,384	184,800	89.54
2020	195,441	175,220	89.65
2021	216,059	196,372	90.89

### The disease spectrum

**Figure 2:**
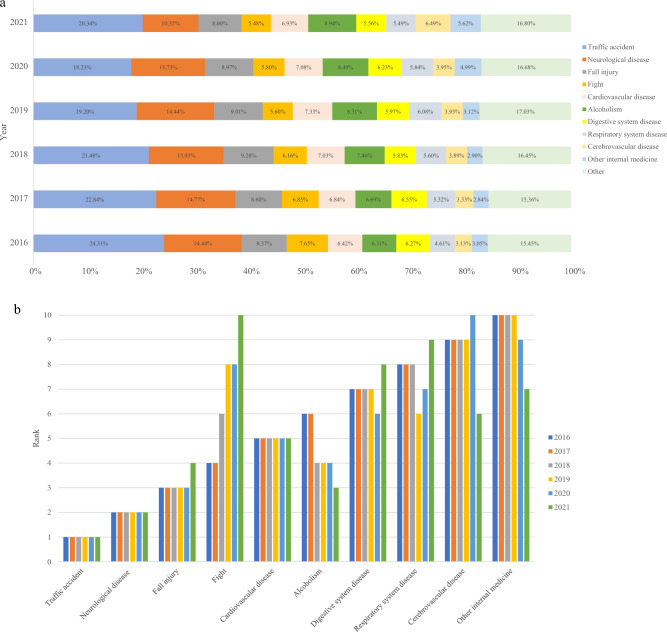
(**a**) The disease spectrum of pre-hospital emergency in the city proper of Chengdu from 2016 to 2021 (**b**) Ranking of the top ten diseases of pre-hospital emergency in the city proper of Chengdu from 2016 to 2021.

From 2016 to 2021, the top ten categories of the disease spectrum were the same, but the order was different (Fig. 2). According to the results, the number of visits for traffic accidents showed a downward trend in 2016–2020 but increased in 2021. Furthermore, we also found an increase in emergency visits for digestive system illnesses in 2020, and cerebrovascular disease increased from 7602 cases in 2019 to 13,771 cases in 2021. However, EMS demand for diseases of the respiratory system declined during the pandemic. Moreover, we found that the distribution of the top ten diseases in different years between 2019 and 2021 was statistically significant ($p<0.01$).

### Circular distribution results of visit months and the top ten diseases

Through the calculation formula of the circular distribution method, we obtained the distribution of monthly prehospital emergency house calls and the distribution of the top 10 diseases in the disease spectrum. In Table 2, we show that the peak periods of emergency visits prior to COVID-19 were early April to mid-February of the following year; the peak periods for 2020 and 2021 were early May to late December and mid-March to late December, respectively. The distribution of the top 10 diseases is shown in Table 3, and following the Rayleigh test, these diseases likewise had peak periods. The onset of the peak periods of traffic accidents, neurological diseases, fall injuries, fight injuries and digestive system diseases was delayed after the outbreak of the pandemic; however, the onset of the peak periods of cardiovascular diseases and respiratory diseases occurred earlier. In contrast, the onset of the peak period of these diseases returned to normal in 2021 or even earlier.

**Table 2:** Circular distribution results of visiting months.

Period	$\overline{a }$	s	P	Peak day	Peak durations
2016–2019	249.09	154.91	< 0.01	Sep.9th	Apr.4th–Feb.13th (next year)
2020	250.77	124.62	< 0.01	Sep.10th	May.7th–Jan.16th
2021	211.54	142.62	< 0.01	Aug.2nd	Mar.11th–Dec.25th

**Table 3:** Circular distribution results of the top ten diseases.

Category	2016–2019		2020		2021	
	Peak durations	p	Peak durations	p	Peak durations	p
Traffic accident	Mar.18th–Dec.25th	< 0.01	May.12th–Dec.16th	< 0.01	Apr.4th–Dec.4th	< 0.01
Neurological disease	Mar.23rd–Jan.16th (next year)	< 0.01	Apr.25th–Jan.13th(next year	< 0.01	Dec.30th–Aug.5th(next year)	< 0.01
Fall injury	Apr.3rd–Jan.1st	< 0.01	May.14th–Dec.28th	< 0.01	Nov.17th–Oct.16th(next year)	< 0.01
Fight	Mar.6th–Dec.5th	< 0.01	Apr.28th–Dec.9th	< 0.01	Apr.17th–Mar.2nd(next year)	< 0.01
Cardiovascular disease	Aug.6th–May.7th (next year)	< 0.01	Dec.22nd–Oct.6th(next year)	< 0.01	Feb.23rd–Dec.24th	< 0.01
Alcoholism	Jun.20th–Apr.13th	< 0.01	May.29th–Jan.8th(next year)	< 0.01	Jan.27th–Dec.10th	< 0.01
Digestive system disease	Mar.25th–Jan.11th (next year)	< 0.01	May.6th–Jan.19th(next year)	< 0.01	Mar.2nd–Dec.20th	< 0.01
Respiratory system disease	Sep.5th–May.2nd (next year)	< 0.01	Aug.7th–Apr.28th(next year)	< 0.01	Jul.11th–May.3rd(next year)	< 0.01
Cerebrovascular disease	Jul.16th–Apr.29th (next year)	< 0.01	Jul.9th–Mar.11th(next year)	< 0.01	Jun.13th–Dec.28th	< 0.01
Other internal medicine	Jul.19th–Apr.24th (next year)	< 0.01	Oct.10th–Jun.14th(next year)	< 0.01	May.25th–Jan.2nd(next year)	< 0.01

## Discussion

It is critical to comprehend the effects of significant public health events, such as the COVID-19 epidemic, on prehospital emergency medical services to ensure proper management of similar outbreaks in the future. Our research made a retrospective descriptive study that explored the changes in the epidemiological characteristics of prehospital emergency services to measure the effect of COVID-19 in the city proper of Chengdu. Our results showed that although there was no large-scale epidemic in Chengdu, people's medical behaviors and willingness were still affected. The demand for prehospital EMSs, prehospital emergency commands and dispatches, ERTs and the disease spectrum changed under the influence of the epidemic. Although the postepidemic normalization period began, the relevant observed prehospital emergency indicators in 2021 were still different from those before the pandemic.

Our results showed a decline in prehospital emergency demand during and after the COVID-19 outbreak, with a marked decline in 2020. This decrease can be explained by the fact that people did not know about the virus and were afraid that going to the hospital would increase the chance of being infected and they could not be treated^19–23^. Some studies pointed out that medical resources were redistributed to fight the virus during the outbreak, resulting in a decline in the ability to solve other medical problems during the outbreak, which in turn led to a decline in the utilization of health care services^8^. After in-depth analysis, we found that the stricter the epidemic prevention and control policy was, the lower the number of emergency calls people made. This may be because strict policies restricted people from going out, which in turn led to a reduction in the incidence of accidents such as traffic accidents, fights, and falls. Although the demand increased in 2021, it has yet to return to prepandemic levels, which means the impact of the outbreak on first aid will be prolonged^22^. In addition, we also found an increase in ERTs in 2020, when the outbreak began, but they were reduced to pre-epidemic levels in 2021. This change may be due to limitations imposed by lockdown restrictions. Moreover, since this was a novel coronavirus, both the general public and medical staff knew little about it, resulting in many protective measures at the time of admission, which may also be one of the reasons for the prolongation of ERTs. The divergent results of our study compared to similar studies may be attributed to organizational disparities in the public's access to acute healthcare ^24^. Additionally, the absence of a large-scale epidemic during the research period could also be a contributing factor. This could be mainly attributed to the prompt implementation of strict epidemic prevention measures by relevant departments, including comprehensive screening of close contacts and large-scale nucleic acid testing for all once infected individuals were identified. Furthermore, our country has stringent quality control indicators for pre-hospital emergency medical services. The Chengdu government mandates that every emergency medical hospital must depart for the accident site within five minutes of receiving the emergency call. Thus, doctors and nurses take measures to minimize the time spent on wearing protective equipment.

Our study also suggested that the pandemic influenced the ranking of the disease spectrum and the distribution of visit months. The overall volume of traffic accidents decreased in 2020 due to the serious and strict safer-at-home and lockdown policies implemented during the domestic epidemic^25,26^. In addition, to reduce the risk of infection, people were more willing to stay at home^27^. In 2021, the volume of traffic accidents increased, which may be attributed to the easing of outbreak restrictions, the lifting of lockdown policies and the return to normalized management of the pandemic. For epidemic prevention and control, people needed to stay at home; they reduced their exercise levels, had irregular diets and changed their way of life, which may have resulted in an increase in digestive system diseases^28^. At the peak of the pandemic, the number of visits for cerebrovascular disease decreased, and it was reasonable for patients with transient/mild stroke symptoms to seek medical assistance less often, possibly because of fear of contracting the virus or declining health care capacity^4^. However, in 2021, after the epidemic leveled off, the number of patients with cerebrovascular disease increased substantially, possibly due to the interruption of regular follow-up visits, failure to take medicine on time and failure to comply with medical orders.

It is worth noting that during the outbreak, the demand for first aid for respiratory diseases decreased compared with that before the pandemic, which may be attributed to the fact that in the early days of the COVID-19 epidemic, China completely carried out social distancing, personal hygiene and masking measures. This finding is consistent with previous reports that showed a substantial decline in the incidence of respiratory diseases in other countries^2,3,5–7,9,29^. However, some studies have also indicated that during the early stages of the outbreak, the number of respiratory disease emergency calls and cumulative incidence rate were higher than before the pandemic^13,29^. This is different from our study, which may be attributed to the longer research period we conducted and partly due to the fact that various countries had not yet entered the strict control stage at the early stage of the pandemic. In addition, due to the demand for epidemic prevention and control and the fear of the virus, people were more willing to delay or avoid seeking medical services, which led us to find that the initial day of the peak distribution of both the visit months and disease were delayed in 2020 when performing the circular distribution analysis. In view of the further understanding of the virus and the effectiveness of epidemic prevention and control policies, people no longer refuse medical treatment for fear of being infected, so the initial daily occurrence of the peak period of diseases returned to normal in 2021 or even earlier.

Finally, this outbreak has not only affected people's normal lives, resulting in a decline in the utilization of medical resources but also affected the epidemiological distribution of prehospital emergency resources. Therefore, our study suggests that relevant departments need to find ways to minimize interference with the treatment and prevention of nonepidemic-related health problems and develop plans on how to return to pre-epidemic levels as soon as possible to guide people to seek medical treatment.

As far as we know, our study is the first to investigate how the local COVID-19 epidemic uniquely affects pre-hospital emergency care in the southwest region of China. Additionally, our study's large sample size and complete data are advantages that reduce statistical errors and improve the results' reliability and authenticity. However, our study also has some limitations. First, some diseases were classified according to a certain system, so the impact of the COVID-19 epidemic on a certain disease could not be analyzed. Second, our study included only one center, resulting in poor generalizability of the conclusions. We hope that a later multicenter study can be conducted, which would add weight to our conclusions. Third, there was no large-scale epidemic in Chengdu, resulting in the impact of the outbreak on prehospital first aid being underestimated.

## Conclusions

In short, the epidemic had a profound impact on prehospital emergency services. In particular, prehospital emergency demand decreased considerably. These results come from a population in China, where the spread of COVID-19 has been relatively well controlled. The epidemic itself has an important relationship with the change in epidemiological characteristics of prehospital first aid. We recommend regular monitoring of the use of prehospital first aid in outbreaks to guide and assess public health responses.

## Methods

### Aim

Our study aim to investigate the impact of the COVID-19 pandemic on the epidemiological characteristics of prehospital emergency treatment in a Chengdu.

### Overview

All data were obtained from the prehospital emergency database of the Chengdu Medical Emergency Center from 2016 to 2021, which included all emergency cases in the city proper of Chengdu during this period. And all methods were performed in accordance with relevant guidelines and regulations. This facility is responsible for answering all emergency calls for critical care in the city proper city of Chengdu, deploying ambulances to take patients to the emergency room, and servicing inhabitants of this region. We analyzed the data on the demand of prehospital EMSs, the command dispatch situation of prehospital emergency services, ERTs and the disease spectrum, especially the changes before and during the COVID-19 pandemic. Although our research focused on the changes in 2019, 2020, and 2021, the data from 2016 to 2018 were also analyzed to assess prepandemic features.

### Exposure

**Figure 3:**
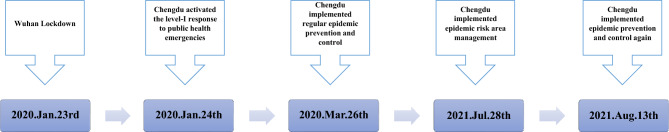
The timeline of Chengdu 's epidemic prevention and control management.

The exposure was Chengdu in 2016 to 2019 (before the pandemic) and 2020 to 2021 (during the pandemic). The timeline of ‘Chengdu’s epidemic prevention and control management illustrates these time periods and related events that occurred during these periods (Fig. 3).

### Exclusion criteria

Repeated calls, cases in which the patient canceled the call for help, cases in which the patient died before the ambulance arrived and cases without complete information, including the passer-by couldn’t give a clear description and the ambulance could not receive the patients were excluded from our study.

### The variables

The prehospital EMS demand included total EMS demand and EMS demand per 100,000 people in the city proper of Chengdu. The population numbers were obtained from the Chengdu Municipal Bureau of Statistics^30^. The disease spectrum was obtained based on the initial diagnoses provided by the attending doctors to the emergency command center after the completion of their mission, and recorded by the emergency center. The command dispatch situation of prehospital emergency services was based on the number of dispatched ambulances and valid dispatched ambulances. The ERT was the time it took for an ambulance to arrive on the scene after an emergency call, which can have a considerable impact on a patient’s survival and prognosis^31^.

### Circular distribution method^32–34^

Circular distribution method uses trigonometric functions to convert the original data into linear data, making it easier to perform statistical analysis and extract more information. We used the circular distribution method to determine the disease’s peak day and peak duration. For populations studying seasonal diseases or other cyclical phenomena, peak periods can be used to represent periods of high incidence within a year. This information has practical significance in formulating disease prevention and control strategies, optimizing agriculture and resource management, and planning for tourism and transportation. Monthly monitoring periods were translated into angles, and the mean angle $\overline{\alpha }$ was used as the concentration of circular distribution data. A year was calculated as 365 days, which equated to 360° of girth, and a day equaled 0.9863°. The median for each month was taken as the median for the group. In January, the median for the group was 15.5 days, converted to 15.2879 degrees. The median for the February group was 45 days, converted to 44.3835 degrees, and so on. Through the principle of trigonometric function substitution, the seasonal peak days and peak periods of the incidence of each disease were obtained, and the calculation formulas were as follows:

$$X=\sum {f}_{i} \mathit{cos}{\alpha }_{i}/\sum {f}_{i} ;Y=\sum {f}_{i}\mathit{sin}{\alpha }_{i}/\sum {f}_{i};$$

$$r=\sqrt{{X}^{2}+{Y}^{2}};s=122.9548^\circ \sqrt{{\mathit{log}}_{10}r};$$

$$\mathit{cos}\overline{\alpha }=X/r;\mathit{sin}\overline{\alpha }=Y/r;$$

$$z=n{r}^{2},$$

where ${f}_{i}$ is the monthly incidence, ${\alpha }_{i}$ is the monthly angle, $r$ is the index of the degree of dispersion of the circular distribution, $s$ is the angle’s standard deviation, which indicates the dispersion of circular distribution data. And Z is the Z value of the statistical result of the Rayleigh test. We used $\overline{\alpha }\pm s$ to estimate peak periods, which covered about 68% of the data with the highest likelihood at the 95% confidence level. When $X>0$ and $Y>0$, $\overline{\alpha }={\mathit{tan}}^{-1}\left(Y/X\right)$; when $X<0$, $\overline{\alpha }={\mathit{tan}}^{-1}\left(Y/X\right)+180^\circ$; and when $X>0$ and $Y<0$, $\overline{\alpha }={\mathit{tan}}^{-1}\left(Y/X\right)+360^\circ$.

### Statistical analysis

We used frequencies (percentages) to express the categorical data and compared the data by the chi-square test. ANOVA was used for quantitative variables. Changes in the EMS demand and ERTs are represented by a line graph. All statistical tests were two-sided, with $P<0.05$ considered statistically significant. SPSS 25 and MS Excel 2021 were used for statistical analysis.

### Ethics approval and consent to participate

This study was approved by the Ethics Committee of the Third People’s Hospital of Chengdu (TPHCD2022S108). The study is for quality improvement purposes and is a retrospective study that involves the review, analysis, and comparison of data from an existing database. Furthermore, participant data were de-identified and aggregated before being transferred to investigators. Informed consent was waived by the Ethics Committee of the Third People’s Hospital of Chengdu.

## Data Availability

The data that support the findings of this study are available from Chengdu Medical Emergency Center but restrictions apply to the availability of these data, which were used under license for the current study, and so are not publicly available. Data are however available from the corresponding author upon reasonable request and with permission of Chengdu Medical Emergency Center.
